# Estradiol Synthesis and Release in Cultured Female Rat Bone Marrow Stem Cells

**DOI:** 10.1155/2013/301540

**Published:** 2012-12-26

**Authors:** Dalei Zhang, Bei Yang, Weiying Zou, Xiaying Lu, Mingdi Xiong, Lei Wu, Jinglei Wang, Junhong Gao, Sifan Xu, Ting Zou

**Affiliations:** ^1^Department of Physiology, Medical College of Nanchang University, Nanchang, Jiangxi 330006, China; ^2^Department of Physiology, Gannan Medical University, Ganzhou, Jiangxi 341000, China; ^3^Institute of Acupuncture and Moxibustion, China Academy of Chinese Medical Sciences, Beijing 100700, China; ^4^Institute of Chinese Minority Traditional Medicine, Minzu University of China, Bejing 100081, China

## Abstract

Bone marrow stem cells (BMSCs) have the capacity to differentiate into mature cell types of multiple tissues. Thus, they represent an alternative source for organ-specific cell replacement therapy in degenerative diseases. In this study, we demonstrated that female rat BMSCs could differentiate into steroidogenic cells with the capacity for *de novo* synthesis of Estradiol-17**β** (E2) under high glucose culture conditions with or without retinoic acid (RA). The cultured BMSCs could express the mRNA and protein for P450arom, the enzyme responsible for estrogen biosynthesis. Moreover, radioimmunoassay revealed that BMSCs cultured in the present culture system produced and secreted significant amounts of testosterone, androstenedione, and E2. In addition, RA promoted E2 secretion but did not affect the levels of androgen. These results indicate that BMSCs can synthesize and release E2 and may contribute to autologous transplantation therapy for estrogen deficiency.

## 1. Introduction

Steroid hormones play important regulatory roles in female reproduction, in which estrogen is essential for folliculogenesis beyond the antral stage and is necessary to maintain the female phenotype of ovarian somatic cells [1–4]. Estradiol-17*β* (E2), a product of androgen aromatization, is the principal estrogen and is secreted in large amounts by the large preovulatory follicles in the ovary [5]. Although the ovaries are the principal source of systemic oestrogen in the premenopausal nonpregnant woman, a number of extragonadal sites of oestrogen biosynthesis, including mesenchymal cells of the adipose tissue and skin, osteoblasts, vascular endothelial, aortic smooth-muscle cells, and brain, become the major sources of oestrogen beyond menopause. However, the total amount of oestrogen synthesized by these extragonadal sites may be small. Within these sites, E2 is probably biologically active only at local tissue level in a paracrine or intracrine fashion without significantly affecting circulating levels [6–8].

The reduction of estrogen production in the ovary may cause menopausal symptoms. In addition, premature ovarian failure may be caused by any process which reduces the number of oocytes within the ovary [9]. For example, chemotherapy can reduce ovarian reserve and affect ovarian stromal function to produce less estrogen [10]. Although estrogen replacement therapy has been established and is recommended for postmenopausal women or patients with hypogonadism, due to its beneficial effects, follicular estrogen production is regulated by a complex set of signals that synergize to produce optimal steroidogenesis [11]. Still, it is difficult to provide an optimal therapeutic dose for long-term estrogen replacement therapy. Furthermore, it is associated with a substantial risk for cardiovascular disease and breast cancer [12]. For this reason, alternative therapies such as steroidogenic cell transplantation may have advantages over HRT for hypogonadism. It should allow control of hormone levels in nature by hypothalamus-anterior pituitary axis.

Several earlier studies have suggested that stem cells can serve as an alternative source for various steroid hormones [13–17]. Bone marrow stem cells (BMSCs) are thought to be multipotent cells, which can replicate as undifferentiated cells and have the potential to differentiate into mature cell types of multiple tissues [18, 19]. In the present study, we investigated whether female rat BMSCs could produce steroidogenic cells with the capacity for the synthesis of E2.

## 2. Materials and Methods

### 2.1. Isolation and Culture of BMSCs

Female SD rats, weighing 80 to 100 g, were obtained from Center of Laboratory Animals of Nanchang University and used in accordance with a protocol approved by the Nanchang University Animal Care and Use Committee. The bone marrow cells were isolated from femurs and tibias of female rats by flushing the shaft with phosphate-buffered saline (PBS) using needles, and the cells were further dispersed several times by gentle, repeated pipetting with a sterile pipet. The dissociated cells were seeded in 75 cm^2^ culture flasks for the primary culture in the high glucose (4.5 g/L) Dulbecco's modified Eagle's medium (DMEM, Hyclone, Utah) supplemented with 10% fetal bovine serum (FBS, Stem Cell Tech Inc., Canada) and incubated at 37°C in a water-saturated atmosphere of 95% air and 5% CO_2_. The nonadherent cells were removed by washing with PBS and replacing the fresh complete medium every 3 or 4 days. The adherent cells were passaged every 7 days by harvesting the cells with 0.25% trypsin/0.02% EDTA, and replating at a 1 : 4 dilution.

### 2.2. Induction of BMSC Differentiation In Vitro

Cultured cells at passage 3 were recovered and used in these experiments. BMSCs were reincubated in 12-well culture plates (Nunc, Denmark) at a density of 2 × 10^5^/well in the high glucose (4.5 g/L) DMEM containing 10% FBS supplemented with or without 10^−5^ mol/L all-trans retinoic acid (RA, Sigma) for 4 days. Differentiated cells from the BMSCs were analyzed by immunocytochemical staining or RT-PCR analysis for expression of aromatase cytochrome P450 (P450arom). The levels of testosterone (T), androstenedione (ASD), and E2 in culture media were measured by radioimmunoassay (RIA).

### 2.3. Immunocytochemistry of P450arom

BMSCs were fixed in 4% acetone at 4°C for 15 minutes and washed 3 times with PBS. Endogenous peroxidase was quenched by incubating the fixed cells with 3% H_2_O_2_ in methanol for 20 minutes. After being washed with PBS for 15 min (5 min⁡×3  times), cells were incubated for 20 minutes with 10% normal goat serum; then with a rabbit polyclonal antibody to aromatase (Boster Co., Wuhan, China), they were diluted 1 : 100 in PBS overnight at 4°C. The negative control was prepared in an identical manner except that the primary antibody was replaced with normal serum. After washing with PBS, cells were incubated with horseradish peroxidase-conjugated goat anti-rabbit IgG for 1 hour at room temperature. After washing, the immunoreaction was detected by using DAB system.

### 2.4. Real-Time PCR Analysis

Total RNA was extracted from cultured BMSCs using Trizol reagent (Sigma, St. Louis, MO) and was reverse transcribed into cDNA using the First-Strand cDNA synthesis kit. Real-time PCR was performed to quantify the samples' cDNA copies using SYBR premix ExTaqTM fluorescent quantitation PCR kit (TaKaRa, Japan). The *CYP19* primers forward: 5′-GCTTCTCATCGCAGSGTAT-3′, reverse: 5′-CAAGGGTAAATTCATTGGG-3′. The *β-*actin primers forward: 5′-GGAAATCGTGCGTGACATTAAA-3′, reverse: 5′-TGCGGCAGTGGCCATC-3′. Conditions for PCR were 40 cycles of 95°C for 5 seconds and 60°C for 34 seconds. The cycle threshold (Ct) was set up at the level that reflected the best kinetic PCR parameters, and melting curves were acquired and analyzed. The 2^−ΔΔCt^ method of relative quantification was used to estimate the copy numbers in *CYP19* gene.

### 2.5. T, ASD and E2 Measurement

Before culture (defined as time 0) and 1, 2, 3, or 4 days after culture, cell culture medium was centrifuged and collected, and the levels of T, ASD and E2 were measured by Beijing Sino-UK Institute of Biological Technology.

### 2.6. Statistical Analysis

The experiment was repeated a minimum of three times. All data were expressed as the mean ± SD and analyzed by ANOVA and Ducan's multiple range test using the SAS 8.0 software. *P* < 0.05 was considered significantly different.

## 3. Results

### 3.1. RT-PCR Analysis for *CYP19* mRNA Expression

**Figure 1:**
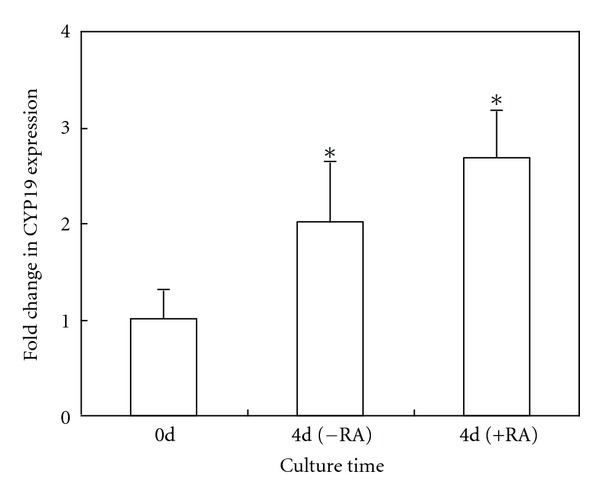
Real-time PCR analysis of CYP19 expression in rat BMSCs. The expression of CYP19 was determined relative to the *β*-actin expression. Expression data (*n* = 5) were reported as fold change (2^−ΔΔCt^). BMSCs cultured in high glucose DMEM alone (control) or in combination with RA treatment (10^−5^ mol/L) for 4 days as compared with 0 day cells. The experiment was repeated three times. Statistical significance was determined by a *t*-test. *P < 0.05, versus 0 day.

RT-PCR analysis showed that there was expression of aromatase gene *CYP19* in BMSCs cultured for 4 days in a high glucose DMEM, and the expression was significantly higher than that in 0 day cells. Furthermore, we investigated the effects of RA on BMSC differentiation in vitro. The result showed that the expression of* CYP19* mRNA was not further elevated by RA treatment at a concentration of 10^−5^ mol/L (Figure 1).

### 3.2. Immunocytochemical Analysis of P450arom

**Figure 2:**
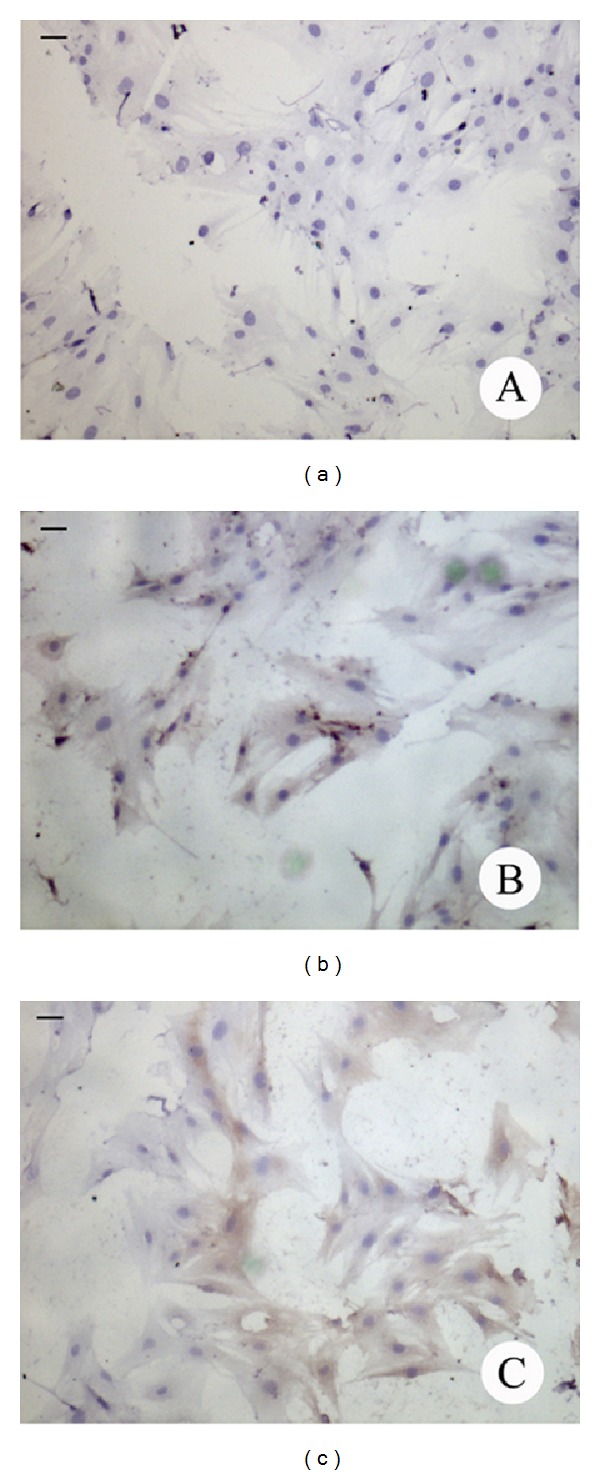
Immunocytochemical staining of P450arom in rat BMSCs cultured for 4 days. (a), negative staining; (b), control group; (c), RA treatment group. P450arom was expressed in BMSCs cultured in a high glucose culture condition alone (control group) or in combination with RA treatment. Scale bar: 20 *μ*m.

To elucidate the capacity of BMSCs to generate E2, we examined the expression of P450arom protein, the enzyme responsible for estrogen biosynthesis, by immunocytochemical staining. The results showed that P450arom was expressed in BMSCs cultured in a high glucose culture condition alone or in combination with RA treatment, with a positive labeling in part of the cells, and it was primarily immunolocalized in the cytoplasm (Figure 2).

### 3.3. RIA for E2

**Figure 3:**
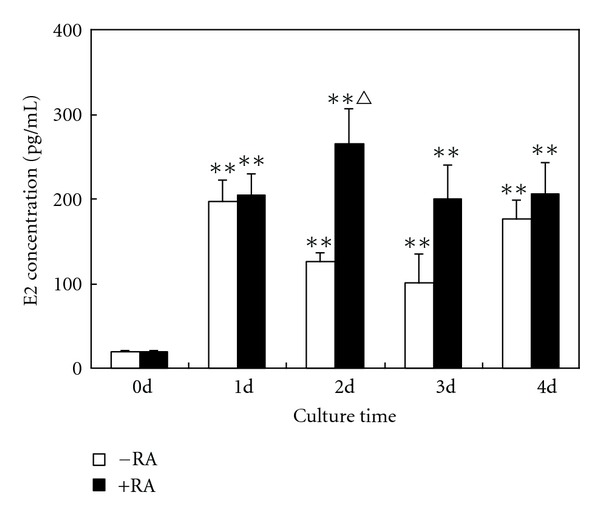
Measurement of E2 concentrations in culture media by RIA. Compared with 0 day cells, the release of E2 significantly increased in rat BMSCs cultured in a high glucose DMEM or in combination with RA for 1–4 days. The experiment was repeated three times. Values represent means ± SD (*n* = 5). **P < 0.01, versus 0 day;  ^△^ *P* < 0.01, versus high glucose DMEM.

To evaluate E2 biosynthesis and release in cultured BMSCs, the levels of E2 in culture medium were measured by RIA. Before incubation, the culture medium of BMSCs at passage 3 contained a low concentration of E2. After exposure to a high glucose condition for 1 day, E2 levels were increased significantly. However, E2 content was not obviously altered after prolonged culture time (2–4 days). Similar results were observed in cultured BMSCs that were given a combined treatment with high glucose and RA. The maximal effect of RA was observed in BMSCs cultured for 48 hours, and the release of E2 significantly increased compared with the high glucose medium alone (Figure 3).

### 3.4. RIA for T and ASD

**Figure 4:**
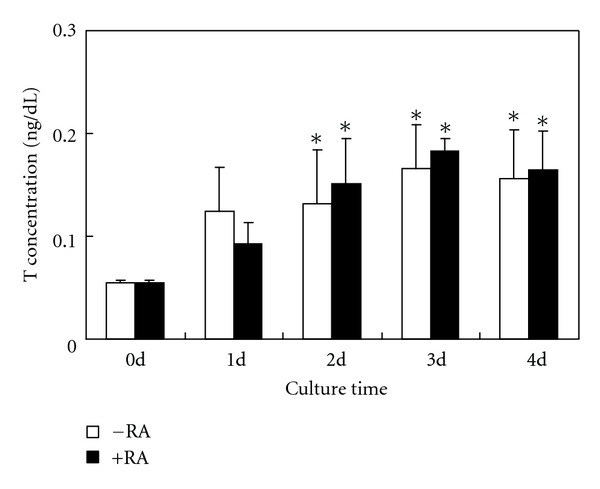
Measurement of T concentrations in culture media by RIA. Compared with 0 day cells, the release of T significantly increased in rat BMSCs cultured in a high glucose DMEM for 2–4 days. RA treatment did not obviously increase the levels of ASD. The experiment was repeated three times. Values represent means ± SD (*n* = 5). *P < 0.05, versus 0 day; **P < 0.01, versus 0 day.

**Figure 5:**
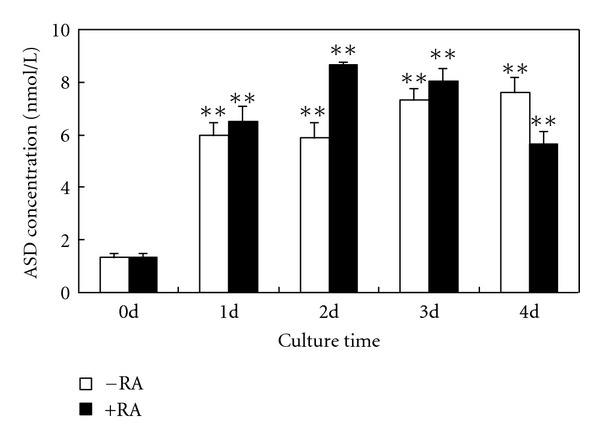
Measurement of ASD concentrations in culture media by RIA. Compared with 0 day cells, the release of ASD significantly increased in rat BMSCs cultured in a high glucose DMEM for 1–4 days. RA treatment did not obviously increase the levels of ASD. The experiment was repeated three times. Values represent means ± SD (*n* = 5). *P < 0.05, versus 0 day; **P < 0.01, versus 0 day.

To investigate *de novo* synthesis of E2, we measured the levels of T and ASD in medium by RIA. After BMSC culture for 4 days and 1 day in high glucose medium alone or together with RA, the release of T and ASD significantly increased, respectively. However, there were no obvious differences in the levels of T and ASD between the high glucose group and combination group (Figures 4 and 5). In addition, prolonged culture time (2–4 d) had no effect on the release of ASD (Figure 5).

## 4. Discussion

The high degree of stem cell plasticity provides a promising strategy for cell replacement therapy. During the past several years, a great deal of attention has been focused on the plasticity of BMSCs. Since BMSCs have tremendous differentiative potential, they can differentiate in vitro and in vivo into mature cells of the heart, liver, kidney, lungs, GI tract, skin, bone, muscle, cartilage, fat, endothelium, and brain. These BMSC-derived cells have been shown to contribute to clinical treatment of genetic disease or tissue repair [20–29]. In the present study, we investigated the ability of the BMSCs to generate steroidogenic cells and release E2 in vitro. Our study revealed that BMSCs cultured in high glucose DMEM with or without RA were capable of differentiating into cells that produced and secreted significant amounts of E2. 

Under physiological conditions, E2 is produced *de novo* from cholesterol and synthesized by the ovary in a sequential manner. Steroidogenic granulosa and theca cells cooperate under gonadotropin control to produce estrogens by stimulating synthesis of steroidogenic enzyme messenger RNAs [11]. In the theca, under the influence of LH, cholesterol is converted to pregnenolone and metabolized through a series of substrates ending in androgen production. Theca cell-derived androgens transported to the granulosa cells of developing follicles, where they are aromatized to oestrogens by P450arom, the product of the *CYP19* gene, which is responsible for conversion of C19 steroids to estrogen [2, 6, 30]. In our culture systems, P450arom mRNA and protein were expressed in BMSCs, which also produced and released T and ASD. These results suggested that BMSCs could produce steroidogenic cells with the capacity for the synthesis of E2. 

In recent years, some studies found that transfection of BMSCs from human and murine with steroidogenic factor 1 (SF-1, an essential factor for differentiation of the pituitary-gonadal axis) can transform BMSCs into steroidogenic cells, which produce various steroid hormones, including E2, and expressed mRNA for P450arom [16, 17]. When transplanted into immature rat testes, adherent marrow-derived cells were found to be engrafted and differentiated into steroidogenic cells that were indistinguishable from Leydig cells [17]. These results provided evidence that BMSCs were capable of differentiating into steroidogenic cells and represented a useful source of stem cells for cell transplantation therapy. In this study, without forced expression of SF-1, we demonstrated the ability of the BMSCs to spontaneously form steroidogenic cells and secrete E2 under a high glucose condition.

RA is well known as the biologically active form of vitamin A and has been shown to play an important role in normal embryonic development and maintenance of differentiation in the adult organism [31]. Previous studies showed that RA could induce BMSCs to differentiate into male germ cells [32] and stimulate E2 and T synthesis in rat hippocampal slice cultures [33]. Therefore, we examined the effects of RA on E2 and T biosynthesis in cultured female rat BMSCs. In the present study, BMSCs were incubated in the absence or presence of RA to investigate RA-induced differentiation of BMSCs to steroidogenic cells in vitro. Compared with high glucose medium alone, E2 secretion was stimulated by RA treatment without any increase in the levels of androgen, suggesting that RA, at least at a concentration of 10^−5^ mol/L, may promote the differentiation of BMSCs to estrogen-producing cells. 

A great deal of efforts had been directed at understanding what role stem cells may play in the physiology and pathology of the mammalian female gonads [34]. Over the past few years, some studies found that bone marrow transplantation (BMT) generated immature oocytes and rescued long-term fertility in a preclinical mouse model of chemotherapy-induced premature ovarian failure. Although all offspring were derived from the recipient germline, donor-derived oocytes were generated in ovaries of recipients after BMT [35]. Furthermore, MSC transplantation can improve ovarian function and structure damaged by chemotherapy, and the paracrine mediators secreted by MSC might be involved in the repair of damaged ovaries [36]. These results suggested that the potential of BMSCs for ameliorating female reproductive function was involved in reversal of both ovarian germline and somatic cell insufficiency.

In a previous report, mouse embryonic stem cells in culture developed into oogonia that could enter meiosis, recruit adjacent cells to form follicle-like structures, which expressed aromatase and secreted E2 [15]. In addition, mouse-induced pluripotent stem cells cocultured with ovarian granulosa cells in vitro could form granulosa cell-like cells and secret E2 [37]. BMSCs in our culture systems did not form follicle-like structures, nor did exhibit the morphology of mature ovarian cells. However, they expressed P450arom, suggesting that BMSCs have the ability to synthesize and to release E2, which may contribute to autologous transplantation therapy of BMSCs for hypogonadism.

## 5. Conclusion

In this study, we showed that female rat BMSCs cultured in high glucose DMEM with or without RA could express CYP19 and P450arom, and excrete T, ASD, and E2. These results indicated that the cultured BMSCs could produce steroidogenic cells with the capacity for E2 synthesis. This study would help to provide basis for clinical application of BMSCs in autologous cell transplantation therapy for patients with estrogen deficiency.
